# Physical activity and sitting time changes in response to the COVID-19 lockdown in England

**DOI:** 10.1371/journal.pone.0271482

**Published:** 2022-07-21

**Authors:** Daniel P. Bailey, Amy V. Wells, Terun Desai, Keith Sullivan, Lindsy Kass

**Affiliations:** 1 Sedentary Behaviour, Health and Disease Research Group, Brunel University London, Uxbridge, United Kingdom; 2 Division of Sport, Health and Exercise Sciences, Department of Life Sciences, Brunel University London, Uxbridge, United Kingdom; 3 Life and Medical Sciences, University of Hertfordshire, Hatfield, United Kingdom; Universitat de Valencia, SPAIN

## Abstract

To reduce the spread of the novel coronavirus disease 2019 (COVID-19), national governments implemented measures to limit contact between citizens. This study evaluated changes in physical activity and sitting in response to the first COVID-19 lockdown in England and factors associated with these changes. A cross-sectional online survey-based study collected data from 818 adults between 29 April and 13 May 2020. Participants self-reported demographic information, physical activity and sitting for a ‘typical’ week before and during lockdown. Participants were grouped into low, moderate and high physical activity, and low and high (≥8 hours/day) sitting. Paired samples t-tests compared physical activity (MET-min/week) before and during lockdown. Pearson’s Chi-squared evaluated the proportion of participants in the physical activity and sitting categories. Logistic regression explored associations of demographic and behavioural factors with physical activity and sitting during lockdown. Walking and total physical activity significantly increased during lockdown by 241 (95% confidence interval [CI]: 176, 304) MET-min/week and 302 (CI: 155, 457) MET-min/week, respectively (P < 0.001). There was a 4% decrease in participants engaging in low physical activity and a 4% increase in those engaging in high physical activity from before to during lockdown (P < 0.001). The proportion engaging in high sitting increased from 29% to 41% during lockdown (P < 0.001). Lower education level (odds ratio [OR] = 1.65, P = 0.045) and higher BMI (OR = 1.05, P = 0.020) were associated with increased odds of low physical activity during lockdown, whereas non-White ethnicity (OR = 0.24, P = 0.001) was associated with reduced odds. Younger age was associated with increased odds of high sitting (OR = 2.28, P = 0.008). These findings suggest that physical activity and sitting both increased during lockdown. Demographic and behavioural factors associated with low physical activity and high sitting have been identified that could inform intervention strategies during situations of home confinement.

## Introduction

The novel coronavirus disease 2019 (COVID-19), caused by severe acute respiratory syndrome coronavirus 2 (SARSCoV-2) infection, was declared as a global health pandemic in March 2020 by the World Health Organization (WHO). To reduce the spread of this highly infectious virus, WHO recommended that national governments implemented measures to limit contact between citizens. The UK government introduced a national ‘lockdown’ on 23 March 2020 during which citizens were confined to their homes, had to follow social distancing measures and were required to self-isolate if they had COVID-19 symptoms [1]. People were only allowed to leave their home for food, health reasons or to work if they were unable to work from home. Schools and leisure facilities were closed and people from different households were not allowed to mix with one another under any circumstances. These restrictions were in place throughout the first phase of the UK’s lockdown, which continued up until 13 May 2020.

Regular physical activity is associated with a significant reduction in the risk of physical and psychological health conditions, such as cardiovascular disease, Type 2 diabetes, some cancers and mental health problems (e.g. anxiety and depression) [2–4]. Higher volumes of sedentary time (i.e. sitting and expending low amounts of energy during waking hours) have also been associated with an increased risk of these adverse health outcomes [5–7]. During the UK’s first national lockdown, opportunities to be physically active were reduced in some respects due to less active travel for commuting purposes, no access to gyms or leisure facilities, and no sporting events taking place. On the contrary, there may have been increased opportunities to be active due to changes in working patterns and spending more time at home. The government also encouraged people to ‘exercise’ at home or once per day outside alone or with members from the same household, which may have increased people’s motivation to engage in physical activity.

The majority of earlier studies reported that physical activity levels had declined and sedentary behaviour had increased during COVID-19 lockdown [8]. An international survey of countries from Europe, North Africa, Western Asia and the Americas found that self-reported walking, moderate, vigorous and overall physical activity (MET-min/week) significantly reduced by 42.7%, 34.7%, 36.9% and 38.0%, respectively, during home confinement compared to before [9]. The number of hours sitting per day also increased by 28.6% [9]. However, previous analysis of the survey conducted in the study presented in this paper found that outdoor cycling and running ≥ 2 times/week increased by 38% during lockdown and walking at least 30-min continuously ≥ 2 times/week increased by 70% [10]. Some evidence suggests that reductions in physical activity occurred predominantly in participants who were physically active before lockdown, whereas physical activity levels were mostly unchanged in individuals who were previously inactive [11]. Other factors such as higher age, low education, being male and being overweight may be associated with greater reductions in physical activity during lockdown [12, 13]. However, there is currently limited data concerning changes in physical activity and sitting during the national lockdown and the factors that may be associated with these changes in people living in England. It is important to address these gaps in knowledge to better inform public health strategies during situations of home confinement, such as the COVID-19 pandemic. The aims of this study, therefore, were to (1) evaluate changes in physical activity and sitting in response to the first COVID-19 lockdown in England, and (2) explore demographic and behavioural factors associated with these changes.

## Materials and methods

### Study design and overview

This was a cross-sectional online survey-based study conducted in England during the country’s first national COVID-19 lockdown. Ethical approval for the study was given by the University of Hertfordshire’s Health, Science, Engineering and Technology Ethics Committee with Delegated Authority (protocol number LMS/SF/UH/04142). Participants provided implied consent by completing and submitting the survey. The survey was active and gathered responses from participants between 29 April and 13 May 2020, which corresponded with weeks 5 to 7 of the first lockdown in England. Citizens were instructed to ‘stay-at-home’ during this phase of the lockdown and were permitted to go outside only for a limited number of reasons. This included shopping for basic necessities, medical needs, for work if people were unable to work from home and for exercise once per day. Leisure facilities were closed meaning people were unable to attend gyms or exercise classes, and organised sports were not permitted. The survey was closed when the lockdown moved into the ‘stay alert’ stage during which more social and family contact was allowed, people unable to work from home were actively encouraged to return to work and a phased re-opening of schools and non-essential retail. The survey was conducted using Qualtrics (QualtricsXM, Provo, USA) and participants were able to click on a link taking them to the survey. Participants were recruited via social media advertising and by emails distributed to University of Hertfordshire staff and students. Individuals were eligible to take part if they were aged ≥ 18 years and resided in England at the time of completing the survey. This study is reported following STROBE guidelines for reporting observational studies [14].

### Measures

The survey had 48 items with questions used and adapted from the Sport England Active Lives survey [15] and the International Physical Activity Questionnaire (IPAQ) [16]. Skip logic was applied for some questions meaning that participants were able to respond to fewer than 48 questions. The survey took an average of 16 min to complete (after removing outliers who did not complete the survey i.e. completed it in less than 8 min or left the survey and returned to complete it more than 60 min later).

#### Demographic variables and body mass index

Information was collected and grouped as follows for the purposes of this study: age (18–39 years; 40–59 years; ≥ 60 years), sex (male or female), education (up to A-level or equivalent; Bachelor’s degree or higher), ethnicity (White or non-White), employment status (unemployed; carer for family member; work from home; furloughed; retired). Participants were asked to self-report their height and weight. Body mass index (BMI) was calculated as: weight / height^2^ (kg/m^2^).

#### Physical activity, sitting and screen time

Physical activity levels and sitting were measured using the IPAQ-short form questions.

The IPAQ is considered to have acceptable validity (median ρ of about 0.3) and reliability (Spearman’s ρ clustered around 0.8) [16]. Participants were asked to answer each question in relation to “BEFORE” and “DURING” lockdown. A custom Microsoft Excel sheet was used to calculate (a) walking, moderate, vigorous and total physical activity MET-min/week before and during lockdown, and (b) physical activity level (i.e. low, moderate and high) before and during lockdown, in line with IPAQ scoring guidelines [17]. For the sitting question, participants reported their sitting time separately for weekdays and weekend days and were able to select < 4 h, 4–6 h, 6–8 h, 8–10 h or > 10 h per day for each question. Participants were grouped as engaging in low (<8 hours/day) or high sitting (≥8 hours/day) before and during lockdown. This threshold was used based on evidence that sitting ≥8 hours/day is associated with a significant increase in the risk of chronic disease and all-cause mortality [18, 19]. Screen time was measured using the question “How much time do you spend on screen-based activities (on any device and excluding work activity) on a typical day during weekdays and weekends?”. Participants could select either < 2 h, 2–4 h, 4–6 h, 6–8 h or >8 h.

### Data analysis

Data analysis was completed using SPSS version 26 (IBM, New York, USA). A complete case analysis approach was used with cases excluded pairwise. Paired samples t-tests were used to compare physical activity levels (MET-min/week) before and during lockdown. Contingency table analysis using Pearson’s Chi-squared test was used to compare the proportion of participants in the low, moderate and high physical activity categories, the proportion of participants engaging in low (<8 hours/day) and high sitting (≥8 hours/day), and the proportion of participants engaging in each screen time category before and during lockdown. Logistic regression was used to explore associations of demographic factors (age, sex, ethnicity and education level), body mass index, and pre-lockdown physical activity and weekday sitting categories with physical activity and weekday sitting categories during lockdown. Models were adjusted for employment status during lockdown, change in total physical activity MET-min/week (for sitting outcome model) and change in weekday sitting category (for physical activity outcome model). Statistical significance was accepted as p ≤ 0.05.

## Results

A total of 818 participants aged 47 ± 13 years completed the survey. All participants provided sitting and screen time data and 790 participants provided complete physical activity data. The descriptive characteristics of the participants is provided in Table 1. The sample was predominantly female and of a White ethnic background. The majority of participants were educated at University level and were working from home at the time of completing the survey.

**Table 1:** Participant characteristics (n = 818).

Characteristic	Category	Percent
Age	18–39 years	30
	40–59 years	53
	≥ 60 years	17
Sex	Male	22
	Female	78
Education	Up to A-level or equivalent	36
	Bachelor’s degree or higher	64
Ethnicity	White	93
	Non-white	7
Employment status during lockdown	Unemployed	10
	Carer for family member	1
	Work outside of the home	12
	Work from home	59
	Furloughed	14
	Retired	4
Body mass index (kg/m^2^)	-	26.2 ± 5.7 (mean ± SD)

### Changes in physical activity levels, sitting and screen time

**Fig 1:**
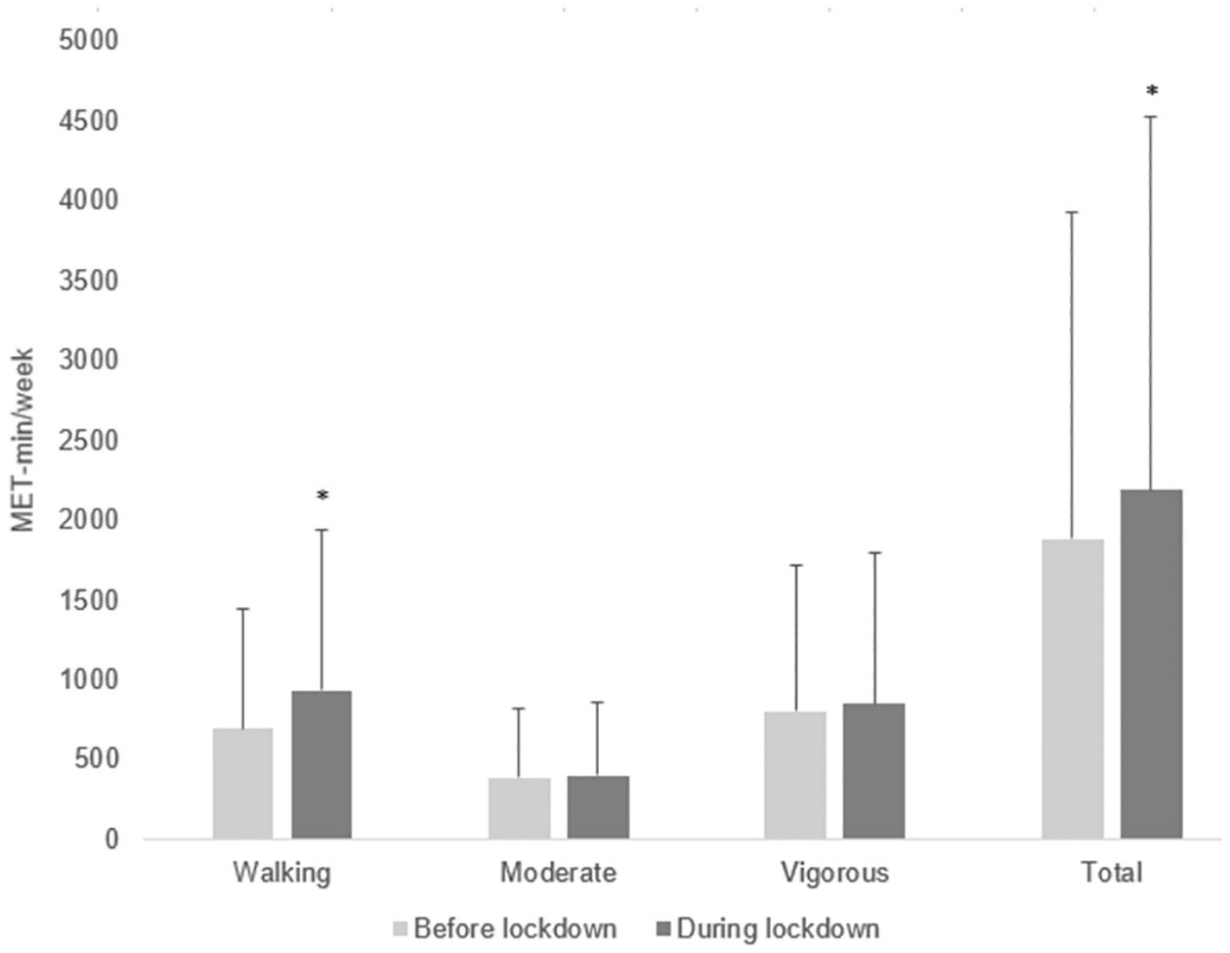
Changes in physical activity before and during lockdown (mean and 95% confidence intervals). * significant difference before compared to during lockdown.

Walking and total physical activity significantly increased during lockdown by 241 (95% confidence interval [CI]: 176, 304) MET-min/week and 302 (CI: 155, 457) MET-min/week, respectively (P < 0.001). Moderate (16 [CI: -34, 63] MET-min/week, P = 0.50) and vigorous physical activity (44 [CI: -49, 139] MET-min/week, P = 0.35) during lockdown was not significantly different compared with before lockdown. Physical activity (MET-min/week) before and during lockdown is shown in Fig 1. The proportion of participants engaging in low, moderate and high physical activity levels is shown in Table 2. Pearson’s Chi-squared was significant, demonstrating that the proportion of participants in the low, moderate and high categories significantly differed before and during lockdown (P < 0.001). There was a 4% decrease (from 26 to 22%) in the proportion of participants engaging in low physical activity, no change in the proportion engaging in moderate physical activity (42% before and during lockdown) and a 4% increase (from 32 to 36%) in the proportion engaging in high physical activity from before to during lockdown.

**Table 2:** Physical activity level and weekday sitting categories before and during lockdown. Data presented as n (%).

		During lockdown				
Sitting (n = 818)		Low	High	Total		
Before lockdown	Low	431 (53%)	152 (19%)	584 (71%)	P < 0.001	
	High	51 (6%)	183 (22%)	234 (29%)		
	Total	482 (59%)	336 (41%)			
		During lockdown				
Physical activity (n = 790)		Low	Moderate	High	Total	
Before lockdown	Low	87 (11%)	81 (10%)	37 (5%)	205 (26%)	P < 0.001
	Moderate	61 (8%)	188 (24%)	80 (10%)	329 (42%)	
	High	22 (3%)	66 (8%)	168 (21%)	256 (32%)	
	Total	170 (22%)	335 (42%)	285 (36%)		

Pearson’s Chi-squared analysis.

**Fig 2:**
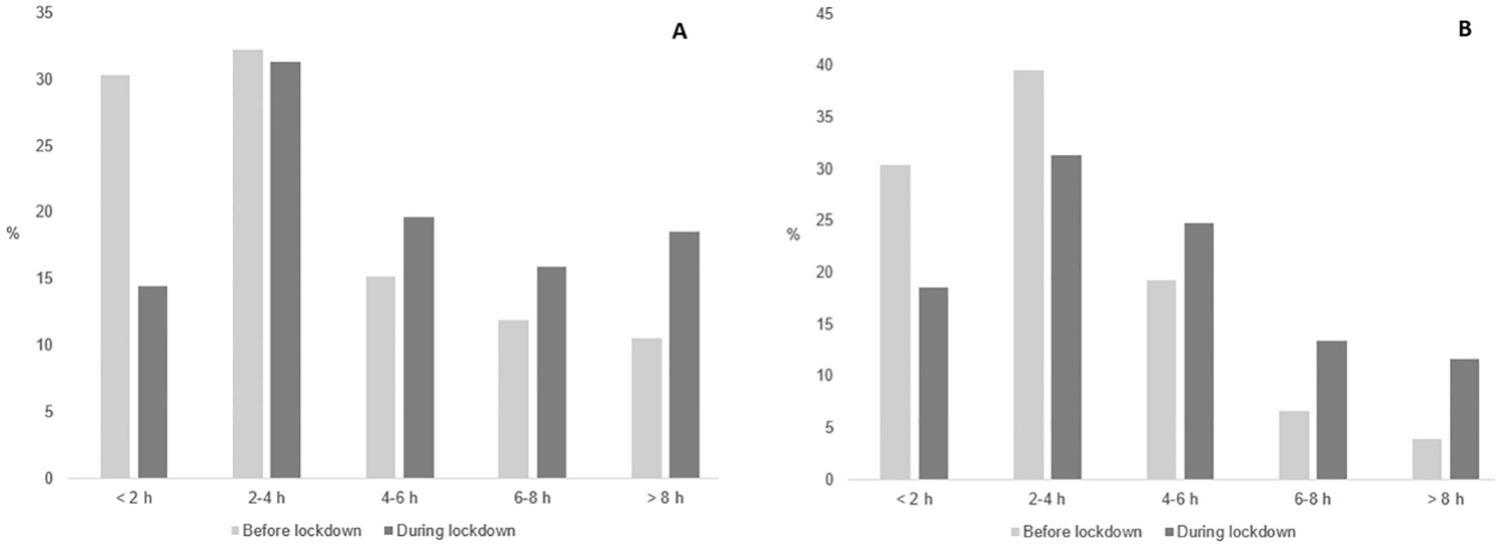
Screen time on weekdays (A) and weekend days (B) before and during lockdown.

The proportion of participants engaging in low and high sitting was significantly different before and during lockdown. There were 22% of participants who engaged in high weekday sitting both before and during lockdown, whereas 53% engaged in low weekday sitting both before and during lockdown. For weekend day sitting, 10% reported high sitting and 73% reported low sitting both before and during lockdown. For weekday sitting, the proportion of participants engaging in high sitting increased from 29% before lockdown to 41% during lockdown (see Table 2). High weekend day sitting increased from 11% to 25% (see S1 Table). As shown in Fig 2, the proportion of participants engaging in higher amounts of screen time (4–6 h and > 8 h) was higher on weekdays and weekend days during lockdown than before lockdown (Pearson’s Chi-Squared P < 0.001).

### Associations of demographic factors and pre-lockdown physical activity/sitting with physical activity and sitting during lockdown

The odds of engaging in low physical activity during lockdown were significantly lower in participants who were non-White compared with White ethnicity (see Table 3). Being educated up to A-level or equivalent was associated with a significantly higher odds of low physical activity compared to those educated to Bachelor’s degree or higher. A higher BMI was associated with significantly increased odds of engaging in low and moderate physical activity during lockdown. There was a trend for higher odds of engaging in low physical activity than high physical activity during lockdown in females compared with males. Participants who engaged in low or moderate physical activity before lockdown had a significantly greater odds of engaging in low and moderate physical activity during lockdown, compared with those who engaged in high physical activity before lockdown.

**Table 3:** Associations of demographic factors and pre-lockdown physical activity with physical activity during lockdown (reference category = high physical activity; n = 711).

Characteristic	Category	Physical activity during lockdown			
		Low physical activity	p-value	Moderate physical activity	p-value
Age	18–39 years	0.93 (0.45, 1.93)	0.852	1.40 (0.78, 2.51)	0.258
	40–59 years	0.71 (0.37, 1.35)	0.708	0.77 (0.46, 1.30)	0.327
	≥ 60 years	Reference category			
Sex	Female	1.70 (0.98, 2.94)	0.060	1.17 (0.74, 1.84)	0.501
	Male	Reference category			
Ethnicity	Non-white	0.24 (0.10, 0.54)	0.001	0.59 (0.27, 1.27)	0.177
	White	Reference category			
Education	Up to A-level or equivalent	1.65 (1.01, 2.68)	0.045	1.34 (0.91, 1.99)	0.142
	Bachelor’s degree or higher	Reference category			
Body mass index (kg/m^2^)	Continuous variable	1.05 (1.01, 1.09)	0.020	1.04 (1.00, 1.07)	0.028
Physical activity before lockdown	Low physical activity	19.81 (10.28, 38.20)	< 0.001	6.06 (3.56, 10.32)	< 0.001
	Moderate physical activity	5.84 (3.15, 10.84)	< 0.001	6.78 (4.41, 10.42)	< 0.001
	High physical activity	Reference category			

Data presented as odds ratio (95% confidence intervals).

Individuals aged 18–39 years had significantly higher odds of engaging in high sitting during lockdown compared with those aged ≥ 60 years (see Table 4). The remaining demographic variables and BMI were not significantly associated with sitting category during lockdown. The odds of engaging in high sitting during lockdown were significantly increased in participants who engaged in high sitting compared with low sitting before lockdown.

**Table 4:** Associations of demographic factors and pre-lockdown weekday sitting with weekday sitting during lockdown (reference category = low sitting; n = 699).

Characteristic	Category	High sitting during lockdown	p-value
Age	18–39 years	2.28 (1.24, 4.18)	0.008
	40–59 years	1.26 (0.72, 2.22)	0.417
	≥ 60 years	Reference category	
Sex	Female	1.06 (0.68, 1.67)	0.792
	Male	Reference category	
Education	Up to A-level or equivalent	0.94 (0.63, 1.42)	0.769
	Bachelor’s degree or higher	Reference category	
Body mass index (kg/m^2^)	Continuous variable	1.01 (0.98, 1.04)	0.574
Sitting before lockdown	High sitting	11.41 (7.45, 17.46)	< 0.001
	Low sitting	Reference category	

Data presented as odds ratio (95% confidence intervals).

## Discussion

The main findings of this study were that self-reported walking and total physical activity significantly increased during lockdown with a simultaneous rise in the proportion of participants engaging in high sitting. Furthermore, novel factors that may explain changes in physical activity and sitting have been identified and could help inform strategies to encourage regular physical activity and limiting sitting time during periods of home confinement.

The increase in total physical activity was largely accounted for by an increase in walking, which could be suggestive of individuals walking more for leisure. Indeed, citizens in England were encouraged to exercise daily during lockdown and were permitted to do this outside of the home once per day. People were also spending more time at home, working less and may have had increased social support from others in their household to engage in physical activity. Collectively, this may have led to an increase in opportunities and motivation to be active [10, 13, 20]. Previous data from the present study’s survey found that never exercising or taking part in exercise classes (online or in-person) decreased during lockdown by 51%, which supports the notion that individuals increased their physical activity by other means such as walking, running or cycling outdoors [10]. This is similar to findings from Israel that demonstrated 70% of individuals trained less than usual during lockdown [21]. Periods of home confinement may, therefore, provide opportunities and motivation to increase physical activity, but this is less likely to be in the form of exercise classes and gym-based physical activity.

In contrary to the present findings, a systematic review found that the majority of previous studies around the globe reported reductions in physical activity levels as a result of their respective lockdowns [8]. This inconsistency could be due to differences in sample characteristics, with some previous studies having a more evenly distributed proportion of females and males and a greater proportion of non-White participants [9, 12]. Nevertheless, there is literature that has reported no change, or decreases, in physical activity in samples with similar demographic characteristics to the present study, such as age, education level and a higher proportion of females [11, 20, 22, 23]. This includes another study of UK adults in which participants reported their average number of steps per day significantly reduced during lockdown [24]. It is difficult, though, to make direct comparisons of this UK based study with the present data in which physical activity was measured as MET-min/week. Similar to the present study, there was a significant increase in daily physical activity time during lockdown in male participants residing in Beijing [25]. However, in the same study, daily exercise time and daily steps significantly decreased in both males and females [25]. This supports the notion that the measurement methods employed may influence the outcomes of these studies. To better inform public health priorities during situations of home confinement and social distancing, it is recommended that future studies attempt to standardise the methods used for physical activity surveillance.

Engaging in low or moderate levels of physical activity before lockdown were associated with a significantly higher odds of engaging in low physical activity during lockdown, which supports previous evidence [26]. Even though physical activity increased across the whole sample, only 4% of participants moved from the low active to high active group, whereas all participants who were moderately active remained in that group during lockdown. This suggests that despite an overall increase in total physical activity MET-min/week, the increase was not great enough to move the majority of the low active participants to moderate or high activity levels. Health promotion strategies are thus needed that focus on increasing physical activity levels of adults during home confinement.

The findings of the present study may be important in identifying population groups that health promotion strategies should be focused towards during home confinement situations. Increasing BMI was associated with higher odds of low and moderate physical activity levels, which supports previous studies [12, 27]. A novel finding was that non-White participants had a reduced odds of engaging in low physical activity during lockdown. This could be due to increased social support for activity from spending more time at home in larger households, or non-White participants’ working patterns being less affected e.g. lower prevalence of being furloughed. However, these suggestions are purely speculative and the reasons for their higher activity level during lockdown compared with White participants requires further investigation. It should also be noted that the sample contained only 7% of participants with a non-White ethnicity, which is likely to be unrepresentative of the general population; these findings should thus be interpreted with caution. A further novel finding was that a lower education level was associated with higher odds of being in the low physical activity group during lockdown. Individuals with a lower education could be less motivated to engage in physical activity due to being less health conscious [28]. They may also have restricted opportunities due to limited access to environments suitable for activity (e.g. more traffic and less supportive for walking) [29] or higher neighbourhood density [30]. However, education level was not associated with physical activity levels during lockdown in Turkish migrant adults living in Germany, or Belgian adults [13, 26]. There was a trend for females to be more likely to engage in low physical activity during lockdown in the present study. Similarly, being female was associated with higher odds of low physical activity during lockdown in Turkish migrant participants living in Germany, [26]. However, in Belgian adults who were categorised as low active before lockdown, sex and education were not significantly associated with changes in exercise during lockdown [13]. This could suggest that health promotion strategies need to be tailored to the needs of different geographical locations, environments and lockdown measures imposed to effectively support increases in physical activity during periods of home confinement and social distancing. Based on the current study, individuals in the UK with a higher BMI, White ethnicity, lower education level and female could benefit most in this respect.

The present study adds to the consistent evidence that sedentary behaviour increased as a result of COVID-19 lockdown [8, 20, 22, 23, 25]. The prevalence of high sitting increased by 12% and 14% on weekdays and weekend days, respectively. The odds of engaging in high sitting during lockdown were significantly higher for individuals who engaged in high sitting before lockdown. This suggests that for individuals whose work routines and leisure pursuits predominantly comprised of sedentary activities, lockdown may not have provided the opportunity or motivation to sit less. The only demographic factor significantly associated with high sitting during lockdown was age; younger adults (aged 18–39 years) had an increased odds of high sitting compared with participants aged ≥ 60 years. Younger adults may have engaged in more sedentary activities for domestic entertainment purposes, such as socialising online and video gaming, than older adults, or may have had more changes in their working situation (e.g. working from home or job loss) [31]. Female sex and high BMI were associated with increased odds of engaging in more sedentary behaviour during lockdown, whereas age was not, in Turkish Migrant German participants [26]. Although there may be inconsistencies regarding the factors associated with increased sedentary behaviour, the available data provides compelling evidence that interventions are needed to address the high volumes of sitting that occur during lockdown. The findings of the present study are important as they identify younger adults as a population group that may especially benefit from interventions to avoid increases in sitting during home confinement and social distancing.

The strengths of this study include extending the limited knowledge regarding factors associated with physical activity and sitting during lockdown in England. This evidence is important for identifying population sub-groups that may particularly benefit from health promotion strategies during periods of home confinement and social distancing. A further strength is the use of a validated questionnaire to measure physical activity levels and sitting time. Limitations of the study include selection bias due to a convenience sample being studied that was not representative of the general population; the sample included over-representation of females and White ethnicity. However, the findings may still be generalisable and help inform health promotion strategies for the higher represented population groups in this study. The self-report nature of the study may have led to overestimations in physical activity and underestimations of sitting [32, 33]. Asking individuals to recall their physical activity and sitting from before the lockdown started may also have led to bias. However, it is hypothesised that this recall bias would be consistent for both before and during lockdown measurements, meaning that changes in physical activity and sitting would not be affected.

In conclusion, there was an increase in physical activity and sitting during lockdown. Adults who engage in lower levels of physical activity and high sitting before lockdown are more likely to engage in low physical activity and high sitting during lockdown. Individuals with lower education, higher BMI and White ethnicity may benefit from interventions to reduce the likelihood of engaging in low physical activity during lockdown. Individuals of a younger age may be targeted to reduce the likelihood of engaging in high amounts of sitting. These findings demonstrate that intervention strategies to mitigate increases in sitting are especially warranted under conditions of lockdown where people are confined to their homes. A range of factors associated with low physical activity and high sitting have been identified and can inform public health promotion interventions during situations of home confinement and social distancing.

## Supporting information

S1 Table Weekend day sitting categories before and during lockdown (data presented as %). (DOCX)
