# Supplementary material for: Physical activity and sitting time changes in response to the COVID-19 lockdown in England
Source: PLoS One. 2022 Jul 21;17(7):e0271482. doi: 10.1371/journal.pone.0271482 (PMC9302792; doi:10.1371/journal.pone.0271482)
Supplement: S1 Table — (DOCX) [file pone.0271482.s001.docx]

**Supplementary Table 1** Weekend day sitting categories before and during lockdown (data presented as %)

|  |  | During lockdown | |  |  |  |
| --- | --- | --- | --- | --- | --- | --- |
| Sitting (n = 818) |  | Low | High | Total |  |  |
| Before lockdown | Low | 600 (73%) | 126 (15%) | 726 (89%) | P < 0.001 |  |
|  | High | 14 (2%) | 78 (10%) | 92 (11%) |  |  |
|  | Total | 614 (75%) | 204 (25%) |  |  |  |
